# What Does Time-Dependent Fluorescence Shift (TDFS) in Biomembranes (and Proteins) Report on?

**DOI:** 10.3389/fchem.2021.738350

**Published:** 2021-10-29

**Authors:** Federica Scollo, Hüseyin Evci, Mariana Amaro, Piotr Jurkiewicz, Jan Sykora, Martin Hof

**Affiliations:** J. Heyrovský Institute of Physical Chemistry of the CAS, Prague, Czechia

**Keywords:** hydration, time-dependent fluorescence shift, biomembranes, calcium, oxidized phosholipids, cholesterol, membrane dynamics, lipid headgroups

## Abstract

The organization of biomolecules and bioassemblies is highly governed by the nature and extent of their interactions with water. These interactions are of high intricacy and a broad range of methods based on various principles have been introduced to characterize them. As these methods view the hydration phenomena differently (e.g., in terms of time and length scales), a detailed insight in each particular technique is to promote the overall understanding of the stunning “hydration world.” In this prospective mini-review we therefore critically examine time-dependent fluorescence shift (TDFS)—an experimental method with a high potential for studying the hydration in the biological systems. We demonstrate that TDFS is very useful especially for phospholipid bilayers for mapping the interfacial region formed by the hydrated lipid headgroups. TDFS, when properly applied, reports on the degree of hydration and mobility of the hydrated phospholipid segments in the close vicinity of the fluorophore embedded in the bilayer. Here, the interpretation of the recorded TDFS parameters are thoroughly discussed, also in the context of the findings obtained by other experimental techniques addressing the hydration phenomena (e.g., molecular dynamics simulations, NMR spectroscopy, scattering techniques, etc.). The differences in the interpretations of TDFS outputs between phospholipid biomembranes and proteins are also addressed. Additionally, prerequisites for the successful TDFS application are presented (i.e., the proper choice of fluorescence dye for TDFS studies, and TDFS instrumentation). Finally, the effects of ions and oxidized phospholipids on the bilayer organization and headgroup packing viewed from TDFS perspective are presented as application examples.

## Introduction

The role of the hydration in maintaining the biological function of biomolecules and biomolecular aggregates is unquestionable (Disalvo, 2015; Biedermannová and Schneider, 2016). Water molecules affect their structure, dynamics and mutual interactions. Although water is a relatively simple molecule being built only from three atoms, its capability to form four hydrogen bonds makes its spatial arrangements in solution extremely complex (Kühne and Khaliullin, 2013). This applies also for the water molecules solvating the biomolecules and biomolecular self-assemblies (Martelli et al., 2020). Huge variety of the achievable hydration motifs bestow the biological entities unique and anomalous properties, which are often supportive for their role and function (Ball, 2008).

In the case of proteins, water interacts with a heterogeneous partner (Levy and Onuchic, 2006). Water molecules can be buried inside the core of proteins employing long residence times which makes them an integral component of protein structure. Such water molecules can be located in confined regions such as internal cavities and active sites being trapped also on a substantially longer time scales than water molecules interacting with the surface of a protein (Russo et al., 2004). Interfacial water molecules are affected by the complex protein topography dynamically interacting with amino acid residues of various chemical compositions and physical properties. This all makes the protein hydration complex and its characterization rather challenging.

Lipid bilayers (core of biomembranes) represent a biomolecular self-assembly essential for the existence of living organisms. Lipid bilayers also show a complex hydration patterns (Disalvo, 2015; Tristram-Nagle, 2015), even when composed of a single lipid species only. In comparison to proteins, such bilayers offer water molecules very limited number of different polar groups for the formation of hydrogen bonds. Biomembranes are self-assembled thanks to the interaction of the amphiphilic lipids with water molecules that stabilize the bilayer structures. Although water presence is an urge for the bilayer existence, its role has often been underestimated in the past when elucidating the membrane structure and function. The complex lipid bilayer was oversimplified and viewed as the rigid nonpolar entity sandwiched by bulk water molecules. Advances in both theoretical and experimental techniques have been gradually revealing the full complexity of water—lipid interactions, putting the bilayer hydration on the merited pedestal. Water not only stabilizes biomembranes *via* hydrophobic effect but also serve as plasticising spacer within the lipid headgroups balancing the free volume in the hydrocarbon chains, which enables the formation of liquid phases. Moreover, hydration enhances the configurational space for additional water and lipid populations which assist for instance the peptide binding (Ge and Freed, 2003).

The closer look on the water distribution along the bilayer normal reveals that majority of water molecules can be found in the interfacial headgroup region (Nagle and Tristram-Nagle, 2000). Three basic types of the interfacial hydration modes have been suggested: evidently free water resembling the bulk; water molecules directly interacting with lipids (Tristram-Nagle, 2015), but also less defined “perturbed” water (Sparr and Wennerström, 2001), whose properties are supposed to be affected by the presence of lipid membranes. Please note, that although the interfacial properties of biomembranes are mostly affected by the headgroup structure and its local hydration and orientation, hydrophobic interactions within the hydrocarbon region considerably modulate the interfacial dynamics as well.

In order to track the degree and nature of the hydration of biomolecules and bioassemblies, manifold of the experimental approaches have been introduced. Each of these methods is unique from the perspective of the length-scale and time-scale of the followed parameters (Biedermannová and Schneider, 2016). Inevitably, the apparent inconsistency among the conclusions based on different approaches may arise, and in fact frequently springs out. To minimize these discrepancies, a critical and explaining reviews are necessary to converge to a consistent picture of the interfacial hydration of the biological molecules and assemblies.

The main topic of this contribution is Time-Dependent Fluorescence Shift (TDFS) (Horng et al., 1995; Jurkiewicz et al., 2012a). TDFS (denoted also as solvent relaxation (SR) or time-dependent Stokes shift (TDSS)) characterize both the dynamics of the hydrated segments of proteins and membranes as well as the level of hydration in the site-specific manner. We would like to point out that there is an extensive literature on the characterization of TDFS in proteins (Pal et al., 2002; Pal and Zewail, 2004; Bagchi, 2005; Li et al., 2007; Bhattacharyya, 2008; Chang et al., 2010; Zhong et al., 2011). Quite a number of these studies were focused on the characterization of the nature of the local water in the hydration shells of proteins by TDFS, including the concept of “biological water.” As explained in the following section that concept based on the suggestion of long-range modification of the structure and dynamics of water around proteins appears nowadays questionable. We believe the discussion of these contributions using TFDS in proteins would need the confrontation with the newer literature on the water shell of proteins, which could be the subject of an independent review. In this mini review, we will focus on the applicability of this technique to study biological systems and on the interpretation of its results, with a strong emphasis on model lipid membranes.

Below, we start with a short overview of the current knowledge about the hydration of biological systems. Further, we shortly describe the theoretical background of TDFS method. Then, we cover the interpretation of the relaxation probed in lipid membranes. We address the applicability of the method and clearly point out its limitations. Description of the TDFS instrumentation as well as the choice of the fluorescent probes is also given.

## What Does One Understand as the “Water Shell” of a Lipid Bilayer; Conclusions From Label-Free Techniques

Before focusing on the principles and applications of the TDFS technique for the investigation of lipid bilayers, it is helpful to discuss the current knowledge on the hydration shell of biomolecules obtained by label free techniques. It is essential to acknowledge that within the last decade advances in molecular dynamics simulations as well as in ultrafast vibrational spectroscopy led to a much clearer picture about how one has to understand the water shell of lipid bilayer. The conclusions drawn were summarized in a seminal review by Laage et al. (2017). We take here the liberty to quote directly from this review: “Starting from the interfacial water layer, the hydration shell assumes the structure of bulk-like water within a few layers, typically less than five layers. Claims of a significant long-range modification of the structure and dynamics of water around biomolecules lack theoretical and experimental evidence.” This finding clarifies what are those three basic types of the interfacial hydration modes mentioned in the introduction: 1) bulk water, 2) water molecules directly interacting with lipids which are imbedded in 3) adjacent water layers. Importantly, these water layers are only a few, typically less than five layers.

Despite that relatively low number of interfacial water molecules, it is undoubtedly accepted that the structure and function of the biological membranes are strongly affected by the dynamic properties of the hydration water layer. Indeed, it plays a pivotal role in transport and signalling functions, mediating membrane-membrane interactions, as well as the ones with ions, DNA or proteins (Disalvo and Bakás, 1986; Hamley, 2000; Berkowitz et al., 2006). This is supported by evidences showing that the number of molecules in the hydration shell influences the properties of the lipid bilayer, such as thickness, area per lipid, melting temperature (Ladbrooke et al., 1968; Gawrisch et al., 1990). Even though above a clear definition of the hydration shell is given, one has to acknowledge an intrinsic variability found in the literature which is dependent on the method used (Laage et al., 2017). Different methods might probe different aspects or concepts of the same issue.

The hydration shell in lipid bilayers is highly oriented, due to the tendency of the water molecules to reach the lowest number of hydrogen bonding configuration as well as electric field created by the oppositely charged choline and phosphate groups (Gawrisch et al., 1990). ATR-FTIR studies have detected perturbation of water through several layers beyond the first one (Arsov, 2015). By using computational approaches, an average value of the hydration shell thickness has been estimated to be around 3.5 Å. The value can be determined as the distance to the first minimum in the radial distribution function (Stirnemann et al., 2013; Duboué-Dijon and Laage, 2014; Fogarty and Laage, 2014; Duboué-Dijon et al., 2016). All-atom simulations have shown that bound water might play an essential role in stabilizing the phospholipid self-assembly, by establishing strong hydrogen bonds and bridging between lipids (Calero and Franzese, 2019).

NMR allows to probe the dynamics of water molecules (Laage et al., 2017). Moreover, it gives information about the water molecular reorientation by monitoring the longitudinal spin relaxation rates of water hydrogen or oxygen isotopes or by measuring the quadrupolar splitting, proportional to water orientation order parameters (Abragam, 1961; König et al., 1994; Ulrich and Watts, 1994; Volke et al., 1994; Wassall, 1996; Berkowitz et al., 2006). Historically, NMR significantly contributed to the current knowledge about the hydration layers, e.g., for PE, PG and PC lipids purified from *E. Coli*, two distinct regions could be distinguished in the relaxation time profile obtained by 2H-NMR. A minimum of 11–16 molecules was found to be part of the first hydration shell, corresponding to a correlation time of 90 ms. Furthermore, an exchange with water molecules not strictly belonging to the first layer has been observed (Borle and Seelig, 1983).

Other representative experimental methods to evaluate water molecules lipid membranes are X-ray and neutron diffraction spectroscopies, specifically the combination of LAXS and neutron scattering from isotropic unilamellar vesicles has been proved to give precise information (Tristram-Nagle and Nagle, 2004; Kučerka et al., 2008). SAXS and SANS are nowadays regularly applied to determine molecular structure at the nanometer scale (Svergun et al., 1998; Merzel and Smith, 2002; Koch et al., 2003; Foglia et al., 2010). Briefly, the combined methods allow to calculate structural features, such as bilayer thickness and area per lipid, which are needed, together with other parameters obtained by specific fitting procedures, to gain information about the number of water molecules. By correcting the equation, insights on the steric number of water molecules, namely headgroup structural water, can be obtained (Tristram-Nagle, 2015). By using the above-described approach, this value was estimated to be between 7 and 8 bound water molecules for phosphatidycholine headgroup (Nagle and Tristram-Nagle, 2000). However, a more comprehensive summary and a related comparison with other experimental techniques can be found elsewhere (Tristram-Nagle, 2015).

QENS and THz TDS are other powerful techniques to study the dynamics of water molecules in the hydration shell, strongly bound to lipid membranes (Swenson et al., 2008; Hishida and Tanaka, 2011; Yamada and Seto, 2020). While QENS probes the hydrogen motions over different length scale by varying the wavenumber, the THz TDS monitors the collective hydrogen-bond distortion by measuring the absorbance in the far infrared frequency range (Laage et al., 2017). Taking advantage of their lower scattering section, hydrogen atoms can be replaced by deuterium ones. The latter renders QENS sensitivity to the specific hydrogen atoms, for instance the water ones and not those belonging to the biomolecules (Laage et al., 2017). THz TDS can measure the dynamics of water molecules around 10^−13^ s (1 THz = 0.16 ps) (Rønne et al., 1999; Yada et al., 2008). NMR and inelastic neutron scattering can investigate time scales up to 10^−9^–10^−11^ s (Swenson et al., 2008; Amann-Winkel et al., 2016), but the rotational relaxation time of bulk water is around 10^−13^ s (Hishida and Tanaka, 2011). As such, NMR and inelastic neutron scattering methods are capable of investigating the water molecules in the first hydration shell, without accessing the dynamics of the long-range water molecules in the hydration shell. By THz TDS in DOPC bilayers 1–4 “irrotational” water molecules per lipid have been identified (Tielrooij et al., 2009). Whereas employing QENS the number of bound water molecule was found to be around five in DMPC bilayers decreasing with temperature, the sum of tight and loose water molecules around 10, and constant within the studied temperature range (Yamada et al., 2017).

Further insights could be gained by using a combination of different techniques, e.g., NMR and neutron diffraction study have showed that water molecules in the hydration shells actually interact with the headgroup of DOPE lipids, specifically with the ammonium group (Rhys et al., 2019). Moreover, combining THz TDS with SAXS, it has been concluded that the thickness of hydration shell in the DMPC membranes is approximately 1 nm and there are around 28 water molecules belonging to this hydration shell (Hishida and Tanaka, 2011). Some of these studies aimed to shed light on the localization of the water molecules composing the hydration layer. It has been shown that the phosphatidylcholine headgroup is strongly associated with water, more specifically, phosphate and carboxyl groups are oversaturated in the number of hydrogen bonding, when compared to the bulk water (Foglia et al., 2010).

It should be highlighted that the here given information on the water shell of lipid bilayers are simply selected examples from a large variety of techniques and does not represent a fully comprehensive overview of the literature. The exact definitions of bound water and the obtained results vary for those techniques. Nevertheless, from those selected examples a rather clear picture of the water shell arises, which we would like to summarize here for a phosphatidylcholine bilayer. A phosphatidylcholine headgroup contains, beside of the three glycerol oxygen atoms and two carbonyls, a positively charged choline and a negatively charged phosphate group. Thus, the phosphatidylcholine offers eight oxygen atoms for establishing hydrogen bonds. Interestingly, the experimentally determined numbers of bound water molecules per lipid molecule are found to be in the same order of magnitude. Further the experimental data suggest the water layers adjacent to those bound water molecules to be thinner than 1 nm and formed by about 20 water molecules or less. Beyond that layers water molecules behave as in the bulk.

While these examples are based on label-free techniques, TDFS requires an introduction of low concentrations of aromatic chromophores to the lipid bilayer, which can disturb the studied system. However, as shown in the next paragraph, TDFS can give distinct information on the changes in hydration and mobility at defined positions within the phospholipid headgroup, with the advantage to the label free techniques of being experimentally simple and inexpensive.

## Basic Principles of Time-dependent Fluorescence Shift

**FIGURE 1:**
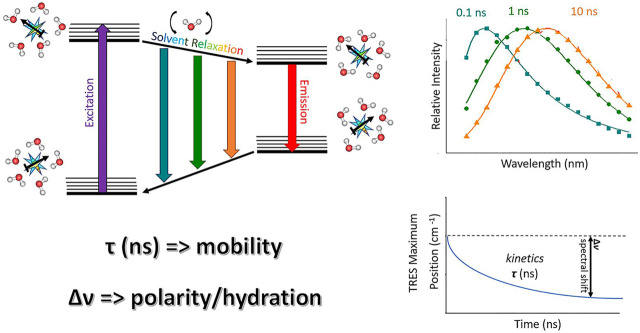
A schematic drawing illustrating TDFS method: On the left, a simplified Jablonski diagram is depicted. A fluorescence probe represented by a light blue star bearing certain dipole moment (black arrow) is solvated by water molecules. These are oriented in the energetically lowest conformation in the ground state. Upon the excitation, a dipole of the dye changes rapidly, organization of the solvation shell thus becomes energetically unfavorable. Consequently, the solvent molecules start to reorient which decreases the energy of the system. This continuous decrease can be accessed by recording TRES which shows red-shift in time (upper right figure). The TRES maxima are then analyzed yielding overall dynamic Stokes shift Δν and characteristic relaxation time τ (lower right figure). Δν and τ reflect the extent of energy relaxation caused by hydration and mobility of the hydrated lipid segments in the probe microenvironment, respectively.

TDFS was originally applied for studying solvation dynamics in neat solvents, e.g., proving water relaxation to occur on the sub-picosecond time scale. It is based on the perturbation of the solvation shell of the fluorophore caused by its rapid electronic excitation accompanied by the change in its charge distribution. Surrounding molecules, when bearing dipole moment, start to reorient in order to adapt to the abrupt change caused by the solute excitation. The solvent molecules keep on rearranging till the energetically favorable state is reached. This continuous relaxation process leading to the dynamic decrease in the energy of the system is known as solvent relaxation. It is projected as the transient red-shift of the recorded time-resolved emission spectra (TRES) being the core of TDFS technique (Figure 1). The TDFS analysis comprise the difference between the energies of initial non-equilibrium Franck-Condon state and fully relaxed excited state, denoted as Δ*ν*, which correlates with polarity of the solvent (Horng et al., 1995). Moreover, the kinetics of the TDFS was found to reflect solvent viscosity (Ito et al., 2004). This is the straightforward interpretation that is applicable in the case of neat solvents. However, the situation gets more complex in the presence of biological molecules and biointerfaces, due to their heterogeneous nature. The most striking difference is the retardation of the TDFS timescales occurring universally for the vast majority of biomolecules and biointerfaces. Specifically, significantly slower components (subnano- and nanoseconds) appear in TDFS kinetics. The explanation for this slow-down implies that the slow TDFS does not originate from the motions of individual water molecules, but is governed by the movements of the hydrated segments of biomolecules. Naturally, the contribution of the segmental movements to TDFS response is highly dependent on the type of biomolecule/bioassembly. Therefore, the analysis and interpretation of TDFS results require profound knowledge of the investigated system. In the following section, we will illustrate this approach on mapping the organization of the lipid bilayers by TDFS, taking into consideration broader atomistic context.

## Time-Dependent Fluorescence Shift in Model Lipid Membranes

### The Origin of the Solvent Relaxation in Lipid Membranes

The molecules of water hydrating lipid bilayer are the major source of polarity sensed by TDFS. The mobility of those water molecules is, however, strongly restricted. Compared to bulk aqueous solutions the timescale of relaxation probed by TDFS slows down from hundreds of femtoseconds to nanoseconds on the distance of 1–2 nm (Sýkora et al., 2002a). Here we focus on the origin of the relaxation process in lipid membranes. In pure water, the relaxation starts with fast librational motions, after which rotations and translations of water molecules establish a new transiently equilibrated molecular arrangement (Horng et al., 1995). Of course, this new order lasts only till the fluorescent emission of the probe occurs. Relaxation in bulk water is affected by the structural properties of the bulk water with its complex, but dynamic, hydrogen bonding network. Spectroscopic data, as well as computer simulations, clearly demonstrate that solutes can affect only the structure of water within a distance of about the size of two to five water molecules and that the disturbance disappears within picoseconds when its source is removed (Zhong et al., 2011; Laage et al., 2017). This is why the structural properties of water are insufficient to explain the three-orders of magnitude slow-down of the relaxation process in lipid bilayers. The frequent exchanges between the molecules of bulk water and those bound to lipids do not contribute considerably to the relaxation process. It is because the arrangement of the newly arrived water molecules is the same as the one being replaced. It is still defined by the arrangement of the molecules of lipids (Horng et al., 1995). Effectively, all water molecules that are entrapped at phospholipid bilayer are bound to lipids and to each other creating a rather well-defined network. In order to dipolarly relax, this network requires movement of the hydrated lipid molecules or their parts. Of course, the method is sensitive only to the molecular motion relative to the fluorophore. This is an important consideration since the molecule of the fluorescent probe can be equally mobile as the molecules of lipids in its surrounding. Because of the above presented arguments, the slow relaxation components observed in a lipid bilayer should be attributed to the movement of the molecules of lipids and fluorophore, rather than the individual movement of the molecules of water (Sýkora et al., 2002a).

### Interpretation of Time-Dependent Fluorescence Shift Results

**FIGURE 2:**
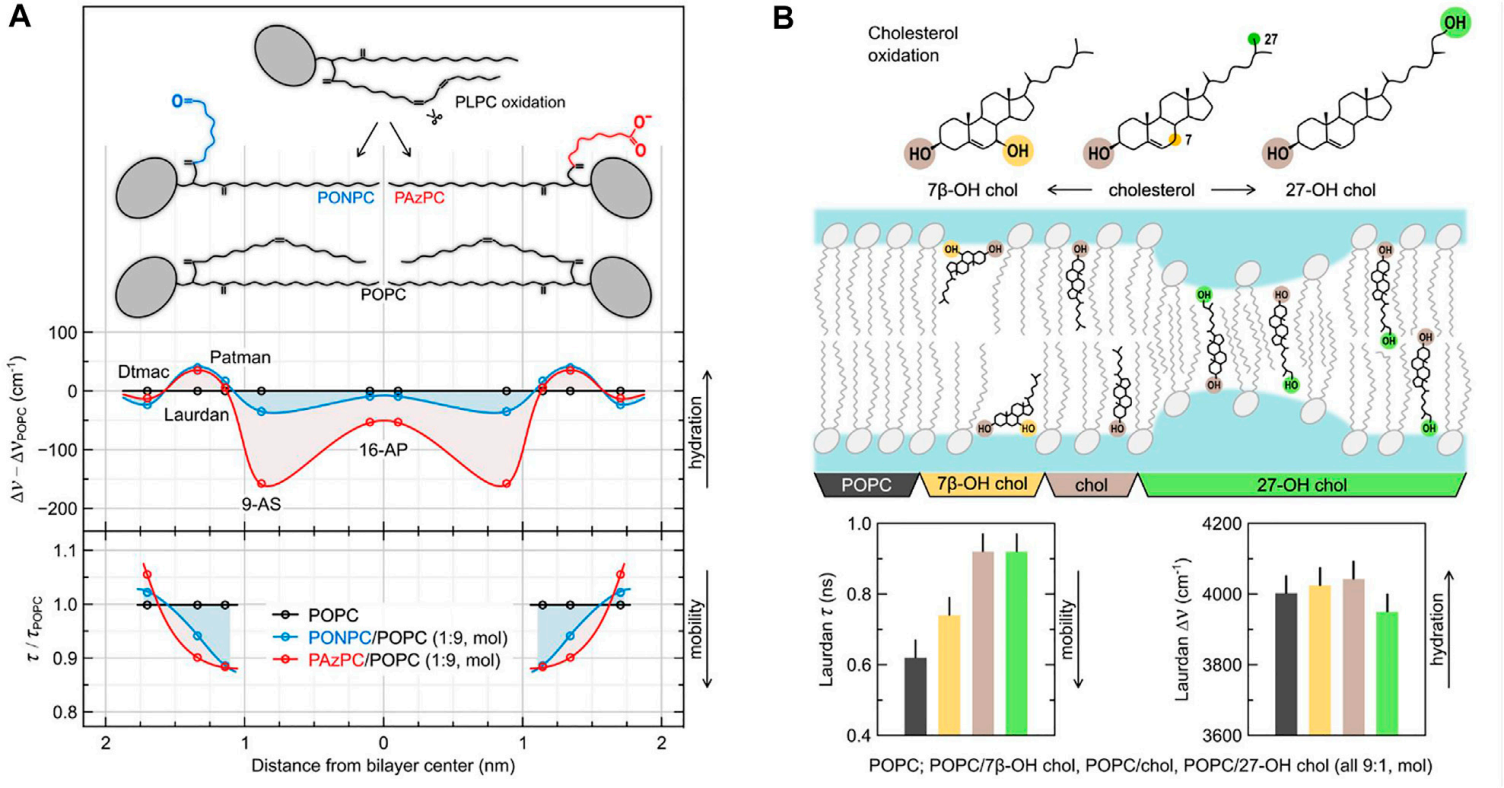
The effects of oxidation of model lipid membranes on local hydration and mobility probed using TDFS. **(A)** depicts the influence of the addition of 10 mol% of truncated oxidized phospholipids (oxPLs) into POPC liposomes. The relative changes in hydration and mobility profiles across the membrane are obtained by comparing TDFS parameters measured in the absence and in the presence of oxPLs. They are presented based on the known or estimated locations of the used fluorophores (Dtmac, Laurdan, Patman, 9-AS, and 16-AP). The schematic structures of the lipid molecules are presented in scale. **(B)** depicts the influence of the addition of 10 mol% of cholesterol and two of its oxidation products into POPC liposomes. Position of the sterol molecules within the bilayer are schematically portrayed based on molecular dynamics simulations, but the effects are exaggerated for the illustrative purposes. The TDFS result shown in the bar graphs are color coded. Experimental details can be found in the original publications the figure was adopted from (Volinsky et al., 2011; Kulig et al., 2015b).

The polarity sensed by TDFS in lipid bilayer is usually determined by the average number of water molecules within the microenvironments of the fluorescent probes. This is the foundation of TDFS sensitivity to membrane hydration. Not only TDFS provides a quantitative measure of membrane hydration (i.e., the extent of energy relaxation caused by hydration), but it also measures it locally (at certain depth within the membrane). This increases the specificity of the obtained information about membrane hydration and allows assessment of the complete hydration profiles of lipid membranes (an example can be found in Figure 2A).

The fact that the nanosecond relaxation probed by TDFS reflects the mobility of hydrated phospholipids is the source of the unique sensitivity of this method to the changes in lipid dynamics. These changes can be easily measured in free standing, fully-hydrated lipid membranes, which is uncommon for other methods including label-free techniques. TDFS allows to study even the subtle effects caused by heavy water (Beranová et al., 2012), specific salt ions (Jurkiewicz et al., 2012b) or lipid and sterol oxidation (Kulig et al., 2015b), to name just a few. As in the case of hydration also the mobility of hydrated lipid moieties is assessed locally. E.g., Patman, which is located at the level of lipid carbonyls, was shown to probe the hydration and mobility of the *sn*-1 lipid carbonyls (Olżyńska et al., 2007). With sensitivity and specificity of TDFS comes the need for the great care when designing and interpreting the results of the experiments.

### Limitations of the Method

TDFS interpretation provided above is strictly valid for deeper locations of polarity probes in lipid bilayer, i.e., when fluorophores are positioned around the level of phospholipid glycerol backbone and deeper. Probes located more outside are to some extend influenced by the fast bulk-water relaxation. This contribution becomes a dominant source of the whole relaxation process for the case the probe is located in the outer part of the headgroup region or further away (Horng et al., 1995). Also, in the case of very deep locations of the fluorophores, proper recording of the whole relaxation process might be impossible (Volinsky et al., 2011). It is due to limited probe lifetime (usually a few nanoseconds) and low polarity caused by only sparse presence of water in this region.

Limited fluorescence lifetime of a probe is also the reason, why TDFS is fully applicable only to lamellar liquid-crystalline lipid phases. Heavily immobilized lipids, e.g., in their gel-phase (S_o_), do not provide sufficient rearrangement during the lifetime of a probe (Amaro et al., 2014). Fortunately, the most physiologically-relevant are the membranes in their liquid phases. The method is applicable not only to liquid disordered (L_d_) phase, but also to the so-called liquid ordered membranes (L_o_). In L_o_ phase the mobility of the molecules is limited by the presence of sterol that fills the gaps between the phospholipids. Nonetheless, for some cases with high content of cholesterol or at low temperatures, limitations of the technique can be also reached in L_o_ membranes (Kulig et al., 2015a).

Fortunately, TDFS methodology provides suitable tools to recognize the problematic situations described above. Careful examination of the full-width at half-maxima (FWHM) of TRES and the time-zero spectrum estimation allow for identification of the number of problems and for evaluation of the completeness of the recorded relaxation process. The basics and applicability of these tools was described in detail in our previous work (Jurkiewicz et al., 2005).

The contribution of lipid molecules to the polarity measured by TDFS is usually limited when compared to that of water molecules. Nevertheless, possible contribution of the system components (including lipids) to the measured polarity, as well as possibility of their specific interactions with fluorescent probe should be evaluated in each case. After the issues related to probe relocation (discussed in section “Choice of the suitable dyes for membrane and protein studies”), the specific influence of the studied molecules on the fluorescent probe are the major source of complicacy in TDFS analysis.

### Steady-State Alternatives for Time-Dependent Fluorescence Shift

The polarity probes utilized in TDFS can be useful even when time-resolved instrumentation is not available. The most common steady-state methods that utilize fluorescent polarity probes and can be applied to characterize lipid membranes include red-edge excitation shift (REES) (Lakowicz and Keating-Nakamoto, 1984; Demchenko, 2002) and generalized polarization (GP) (Parasassi et al., 1990; Bagatolli, 2012). Lower cost of instrumentation, often simplified analysis and faster measurements, but also compatibility of some of them with fluorescence microscopy are advantages that should not be overlooked. In the case of rigorous evaluation of the lipid membrane properties their major drawback is the lack of the kinetic data. Lipid hydration and mobility, the two parameters, that are readily available from TDFS analysis, remain inevitably coupled in steady-state methods, and additional information is needed in order to separate their contributions to the measured steady-state parameters. The absence of such information might lead to certain misinterpretations, e.g., when the changes of a single parameter, like GP, are interpreted exclusively in terms of either hydration/polarity or mobility/fluidity of lipid bilayer, without considering that they may change together and sometimes even compensate for each other (see, e.g., Amaro et al., 2014). A wise compromise between TDFS and the simplified approaches is sometimes possible. For example, the time-resolved version of GP, proposed already by Parasassi et al. (1986), was recently adapted to confocal microscopy allowing successful GP-FLIM analysis of living cells in a manner assuring both sufficient precision and speed needed for live-cell imaging (Ma et al., 2018).

## Interpretation of Time-Dependent Fluorescence Shift in Complex Biomembranes and Proteins

As illustrated above, TDFS possesses a great potential to map hydration and packing effects in monophasic model lipid membranes providing interpretable outputs. The homogeneous membranes present an anisotropic system along the membrane normal, yet staying isotropic in the lateral directions. Although the lipid and probe fluctuations along the z-axis cannot be excluded (Nagle and Tristram-Nagle, 2000), the averaging of the TDFS response over the distribution of locations provides a reasonable estimate on the membrane organization at the bilayer region corresponding to the time-averaged position of the probe. The problem arises at the situations with the multiple probe locations, which were observed for instance for the complex phase separated model bilayers. Most of the common probes partition in both phases differing in TDFS traces. To separate the time courses could be possible by decomposing the multi-component time resolved emission spectra (TRES) into the respective contributions. However, this type of data processing seems to be ill-defined for the limited number of data points in recorded TRES (typically, TRES are recorded in 10 nm steps).

Even more entangled situation in comparison to the complex lipid membranes arises in proteins, which form an anisotropic entity with high chemical heterogeniety in all three dimensions. This results in an extremely complex TDFS response, which was illustrated for example for the dehalogenase enzymes (HLDs). The tunnel mouth region of the selected HLDs was labeled with a set of various fluorophores, based on Coumarin-120 and Prodan-like probes (denoted as Muc) (Amaro et al., 2013). In spite of the fact that the probes were positioned at the same well-defined location, the TDFS response was largely heterogenous. For the Coumarin-based probe, the TDFS kinetics in HLDs appeared to be significantly faster than those obtained for HLDs labeled with *Muc* probes. These experiments revealed that the specific contact sites between the probe and hydrated amino-acid residues (which naturally will be dependent on the chemical structure of the fluorophore) has to be regarded for the quantitative TDFS application. To identify these dye-specific effects the assistance of MD simulations is of high importance.

## Choice of the Suitable Dyes for Membrane Studies

The proper choice of the fluorescent dye is essential for the successful TDFS implementation. The ideal probe should fulfill three basic requirements:

1. In order to introduce sufficient perturbation by the dye photoexcitation into the system, the probe must show a significant difference between the excited and ground state dipole moments in magnitude and/or direction. This difference is usually translated into the large Stokes shift observed for polar solvents.
2. The ideal TDFS dye is to possess a linear solvatochromic behavior, i.e., no specific interactions should interfere with the polarity dependence.
3. The knowledge of the location of the probe is clearly of utmost importance for the correct interpretation of data. A probe should have as defined location in the system of interest as possible, be it a lipid bilayer (e.g., depth of location) or a protein (e.g., specific labelling).

Coumarin-153 is one of the best examples of fluorescent dyes appropriate for TDFS studies, especially for the neat solvents (Horng et al., 1995) and micellar systems (Sonu and Saha, 2016). It shows large Stokes shift and linear solvatochromic behavior. Unfortunately, its derivatives tailored for the protein and membrane labelling are not commercially available which limits its applicability. Nevertheless, other coumarin derivatives, 6,7-dimethoxy-coumarin (Jesenska et al., 2009) and coumarin-120 (Amaro et al., 2013; Stepankova et al., 2013; Sykora et al., 2014), were used for TDFS studies of Dehalogenases. These dyes do not possess as superior characteristics as Coumarin-153, but they enable selective labelling of the biologically relevant region of dehalogenase enzymes. This illustrates that the choice of the dye is often a search for the optimal compromise among the basic requirements listed above. Please note, that not all coumarin derivatives are suitable for TDFS. For example the 7-hydroxy-4-methylcoumarin can adopt different forms resulting in a complex excited state kinetics (Choudhury et al., 2008; Choudhury and Pal, 2009; Amaro et al., 2015) that hinders interpretation of the TDFS data. Nonetheless, this coumarin is still sensitive to the extent of local hydration and can be used for qualitative studies of proteins in a site-specific fashion (Amaro et al., 2015). Similarly, the water-driven proton transfer of tryptophan analogues can be used for mapping hydration in a site-specific manner in biological systems via fluorescence techniques (Shen et al., 2013; Chen et al., 2016). Yet TDFS applicability of those Trp-based dyes is disqualified due to their complex photophysics.

Popular strategy for designing the TDFS probes is the insertion of an electron donating and electron withdrawing groups at a certain distance. This promotes intramolecular charge transfer upon excitation, thus inducing large changes in the dipole moment. The family of amino-substituted naphthalene probes designed in this way have been proven to be highly sensitive to polarity and have been common choices for TDFS studies in proteins and biomembranes. For the latter system, Prodan (Hutterer et al., 1998; Jurkiewicz et al., 2006; Först et al., 2014) and its derivatives Laurdan (Jurkiewicz et al., 2012b; Jurkiewicz et al., 2012c; Först et al., 2014; Macháň et al., 2014; Melcrová et al., 2016; Melcrová et al., 2019) and Patman (Hutterer et al., 1998; Olżyńska et al., 2007; Jurkiewicz et al., 2012c) are used to study the headgroup and carbonyl regions (Jurkiewicz et al., 2006; Jurkiewicz et al., 2012a). Furthermore, the list of the applicable TDFS dyes located in particular areas along the z-axis of a lipid bilayer can be expanded to provide detailed information on membrane hydration and polarity gradient (Hof, 1999; Sýkora et al., 2002a; Jurkiewicz et al., 2005). The examples are, from shallower to deepest, the probes Dauda (Sýkora et al., 2002a), C17DiFU (Sýkora et al., 2002a), DTMAC (Jurkiewicz et al., 2012c; Först et al., 2014; Melcrová et al., 2019), ABA-C15 (Sýkora et al., 2002b), Prodan (Hutterer et al., 1998; Jurkiewicz et al., 2006; Jurkiewicz et al., 2012a; Först et al., 2014), Laurdan (Jurkiewicz et al., 2006; Jurkiewicz et al., 2012a; Jurkiewicz et al., 2012b; Jurkiewicz et al., 2012c; Först et al., 2014; Macháň et al., 2014; Melcrová et al., 2016; Melcrová et al., 2019), Patman (Hutterer et al., 1998; Jurkiewicz et al., 2006; Olżyńska et al., 2007; Jurkiewicz et al., 2012a; Jurkiewicz et al., 2012c), 2-AS (Hutterer et al., 1997; Sýkora et al., 2002a), 9-AS (Hutterer et al., 1997; Sýkora et al., 2002a; Jurkiewicz et al., 2012c), 16-AP (Hutterer et al., 1997; Jurkiewicz et al., 2012c). The use of identical fluorophores located at different positions allows for direct comparative studies to be performed (Jurkiewicz et al., 2012a; Jurkiewicz et al., 2012c). Chemical structures and fluorophore depth of location of these probes can be found in (Jurkiewicz et al., 2005). Since the fluorescent probes in lipid bilayer are relatively mobile, it is especially important to exclude the possibility of their relocation during the experiments. Considering the large gradient of the measured TDFS parameters across the membrane, even relatively small fluorophore instabilities can provide misleading output (Jurkiewicz et al., 2006). Therefore, probe location should be carefully checked by critical analysis of the TDFS results, but also by additional experiments, e.g., quenching experiments (Abrams and London, 1993).

Aminonaphtalene “Prodan-like” dyes have also been utilized for site-specific labelling of proteins for TDFS studies (Guha et al., 2005; Koehorst et al., 2010; Amaro et al., 2013; Cohen et al., 2016; Fischermeier et al., 2017). For example, Badan has been used to probe local protein hydration and dynamics at the active site of copper-transporting ATPase—LpCopA as a function of membrane lateral pressure (Fischermeier et al., 2017). Badan labelling was also applied for the membrane-embedded M13 coat protein to map TDFS at different depths of lipid bilayers (Koehorst et al., 2010). Another example is the use of TDFS of the Prodan-like dye MUC7 to study the hydration and mobility of the tunnel mouth of Dehalogenase proteins (Amaro et al., 2013).

The importance of the choice for the proper TDFS probe can be also strengthened by the inspection of the dyes which are unsuitable for TDFS. For example, NBD dyes display a small change of dipole moment upon electronic excitation of ∽2 D (Amaro et al., 2016). Moreover its solvatochromic behaviour is far from being linear varying randomly with polarity and is sensitive to prominent specific solvent effects. Specifically, hydrogen bonding shifts the emission spectra to lower energies (Fery-Forgues et al., 1993). Therefore, NBD is not a good candidate for TDFS studies and its response in lipid bilayers (e.g., for the headgroup labelled NBD-PS) has been shown to correlate with dye position and water density along the bilayer normal, and not with the environment dynamics (Amaro et al., 2016). The lipophilic Di-4-ANEPPDHQ dye serves as an analogous example. Although it has been used as a reporter of membrane dynamics (i.e., phase state) (Jin et al., 2006; Amaro et al., 2017; Sezgin et al., 2017) it displays a complex TDFS behaviour. The evolution of the TRES FWHM displays multiple maxima recorded in cholesterol containing bilayers that suggest the existence of several underlying relaxation processes. These undesired effects may derive from different phenomena including interactions between the dye and membrane components, specifically cholesterol, or the multiple location of the dye in the membrane (Amaro et al., 2017).

## Time-Dependent Fluorescence Shift Instrumentation

As mentioned above, TDFS response can cover a huge time-span ranging from hundreds of femtoseconds up to microseconds. In addition to the fluorescence lifetime of the probe, which strictly sets the experimental time window, the choice of the instrumentation has to be also considered.

For mapping the ultrafast relaxation processes (i.e. shorter than ∼20 ps), the techniques employing the ultrafast femtosecond lasers combined with the up conversion approach (Chosrowjan et al., 2015), Streak camera detection (Liu et al., 2014) or Kerr gating (Arzhantsev and Maroncelli, 2005) are of the most common choice. These techniques show an excellent time resolution (down to 200 fs for the upconversion and Kerr gating, ∼1 ps for Streak camera set-ups), yet at the expense of the higher excitation powers, elevated instrumental complexity and cost, and certain limitations in the recording of the longer timescales.

Since the relaxation in biosystems are often dominated by the slow nanosecond components the ultrafast kinetics can be often neglected. The instrumentation of choice in such case would be time-correlated single photon counting (TCSPC) and multifrequency domain fluorometry (Boens et al., 2007; Lemmetyinen et al., 2014). Although based on different principles, both techniques yield fluorescence lifetimes with the comparable precision and time resolution (∼10–20 ps) even for multi-component data (Lakowicz et al., 1984) which are typical for TDFS. Moreover, with the advances in semi-conductor laser sources covering substantially UV/VIS region and advances in timing electronics, the instrumentation is financially accessible and easily operable. The time-scale spanned by these techniques usually covers a substantial portion of the TDFS response in the headgroup and hydrocarbon regions of biomembranes and often also TDFS for dyes attached to proteins, which makes the TCSPC and phase-modulated fluorometry an ideal choice for monitoring TDFS in biological systems.

## Time-Dependent Fluorescence Shift in Model Lipid Membranes—Application Highlights

TDFS is nowadays a well-established method for studying model lipid membranes. As discussed before, it is sensitive to membrane hydration and to mobility of lipids. Since these two parameters are easily affected by number of biologically relevant processes, the method can be successfully used to sense lipid packing, phase state of lipids, adsorption of peptides, specific effects of adsorbed salt ions, and oxidation of membrane components, to name just a few of them. Below we present applications that exemplify the sensitivity of TDFS to 1) model lipid membrane oxidation and 2) interaction with calcium ions.

### Oxidized Model Lipid Membranes

Oxidation of membrane components can considerably change both their mobility and hydration (Jurkiewicz et al., 2012c). Phospholipids with polyunsaturated hydrocarbon chains easily oxidize to molecules with polar groups introduced at the end of the truncated hydrocarbon tails (see Figure 2A) and short-chain byproducts (e.g., hydroxynonenal (Fruhwirth et al., 2007; Vazdar et al., 2012). The shortened lipid tail of such an oxidized phospholipid (oxPL) not only changes the shape of the molecule from cylindrical to conical, but also, due to its hydrophilic character, loops back to the headgroup region (Sabatini et al., 2006). This behavior has number of consequences for the structure and dynamics of the lipid bilayer (Beranova et al., 2010; Khandelia and Mouritsen, 2009). First of all, it further increases the spontaneous curvature of the oxPL, which can lead to membrane destabilization, especially in the absence of cholesterol (Khandelia et al., 2014; Štefl et al., 2014). The newly introduced polar moiety can also serve as a carrier for other lipid headgroups lowering the energetic barrier of their flip-flop (Volinsky et al., 2011). TDFS studies, in which we used a series of polarity probes, showed that the introduction of truncated oxPLs changes the whole hydration profile of the lipid bilayer (Figure 2A). Interestingly, the augmented lipid mobility (shorter relaxation times) probed by Laurdan and Patman in oxPL-containing membranes match the faster lateral diffusion of phospholipids measured by fluorescence correlation spectroscopy (FCS) in those systems. This general rule of the correspondence between the time of TDFS relaxation and the time of lateral diffusion of lipids was frequently observed; see e.g., (Olšinová et al., 2018). There are however certain differences between the lateral diffusion of lipids and the local mobility probed by TDFS. For example, as depicted in Figure 2, the truncated oxPL tails with –(C=O)H terminus create voids in the headgroup region of the membrane. These voids allow for much faster relaxation probed by TDFS, but they do not influence the lateral lipid diffusion as much as oxPLs with terminated with –(C=O)O− group (please compare with the FCS results in Beranova et al. (2010). This example shows that probing locally, while having number of advantages, can also lead to misinterpretation. In general, one should avoid direct links between locally measured parameters (such as TDFS relaxation time) and macroscopic parameters (such as viscosity).

Not only phospholipids, but also cholesterol oxidizes. Many oxysterols are products of oxidative stress, but some of them are created by our organisms on purpose to be used in signaling and/or in cholesterol homeostasis (Kulig et al., 2016). Addition of as little as single OH group to cholesterol molecule can strongly influence the properties of a cholesterol-enriched model lipid membranes. It is substantial where this additional OH group is introduced. Based on TDFS measurements and computer simulations of we have distinguished two groups of oxysterols: ring- and tail-oxidized ones (Kulig et al., 2015b). Figure 2B depicts representatives of these two groups: 7β-OH-chol (ring-oxidized) and 27-OH-chol (tail-oxidized). Replacement of cholesterol in POPC lipid bilayer by 7β-OH-chol impairs cholesterol function as membrane stabilizer (cholesterol causes lipid ordering and condensation), which in TDFS is observed as increased mobility of lipids in the presence of 7β-OH-chol. On the other hand, 27-OH-chol does not influence TDFS parameters. But, due to the presence of hydrophilic moieties on both sides of the molecule, 27-OH-chol was found in simulations to oscillate fast between the two lipid leaflets. This movement results in increased membrane permeability to water and small polar solutes, even though, the average hydration probed by Laurdan is unchanged. This property of tail-oxidized sterols is utilized in our bodies. Since cholesterol do not pass through the blood-brain barrier, its excess is eliminated in the form of tail-oxidized sterol (mostly 24-OH-chol). It is advantageous that such oxysterol passes fast across the membranes without disturbing their properties.

### Adsorption of Calcium Ions to Model Lipid Membranes

**FIGURE 3:**
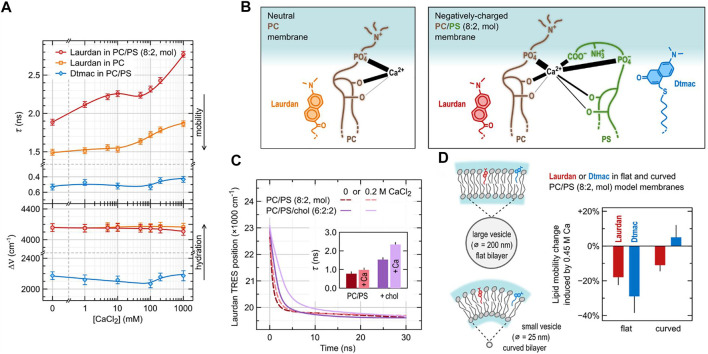
Adsorption of calcium ions to model lipid membranes investigated using TDFS. **(A)** depicts TDFS parameters obtained for unilamellar liposomes composed of either pure phosphatidylcholine (PC) or its mixture with 20 mol% phosphatidylserine (PS) dispersed in CaCl_2_ solutions. Laurdan and Dtmac were used to probe lipid carbonyl and phosphate regions, respectively. **(B)** summarizes the complex calcium interactions with phospholipids as obtained from the joined molecular dynamics and experimental study (Melcrová et al., 2016). Positions of the functional groups of lipids are taken from density profiles obtained in molecular dynamics simulations. The thickness of the black lines joining the functional groups with Ca^2+^ corresponds to the mean number of contacts between them. **(C)** shows the influence of cholesterol (chol) on the calcium adsorption to PC/PS model membranes. The results include the position of the maxima of Time-Resolved Emission Spectra (TRES) of Laurdan together with mean relaxation times (inside) obtained for PC/PS (8:2) and PC/PS/chol (6:2:2) lipid composition in the absence and in the presence of CaCl_2_. **(D)** presents the effect of PC/PS membrane curvature on the adsorption of Ca^2+^.

Various salt ions are omnipresent in cytoplasm and extracellular fluids. In our membranes there are receptors and channels specifically recognizing them, though, their adsorption to lipid bilayer itself is often overlooked. We proved that TDFS can be successfully used to study even subtle effects of the adsorption of salt ions to model lipid membranes (Jurkiewicz et al., 2012b; Vácha et al., 2010). This is because ions often interfere with the network of hydrogen bonds within the lipid headgroups altering mobility of lipids. Electrostatic interactions have an important role in the adsorption of ions, but the specificity of ionic effects is governed by other contributions, e.g., ion polarizability and its affinity to the hydrophilic/hydrophobic interface (Pokorna et al., 2013). In general, cations are attracted to negatively charged membranes as anions are attracted to positively charged ones, but the specific binding sites are determined by other factors. For example, adsorption of Ca^2+^ to the mixed lipid bilayer composed of 80 mol% zwitterionic POPC and 20 mol% anionic POPS is definitely enhanced by the presence of POPS, but the major Ca^2+^ binding sites are not the negatively charged carboxylic groups of POPS. TDFS results based on Laurdan and Dtmac probes, sensing lipid carbonyls and phosphate groups, respectively, together with sum frequency generation measurements, and molecular dynamics simulations revealed a complex picture of calcium adsorption to lipids (Melcrová et al., 2016). Multiple binding sites (lipid carbonyls and phosphates of both POPC and POPS molecules, as well as POPS carboxylic moieties) were occupied in proportions depending on lipid composition and calcium concentration. Ca^2+^ ions were also able to simultaneously bind multiple sites belonging to different lipid molecule bridging them together, which resulted in considerable lateral compression of the membrane and reduced lipid mobility. In TDFS, this was observed as an increase of the relaxation time. TDFS results together with the scheme of complex Ca^2+^-lipid interactions are shown in Figures 3A,B. It is worth noting, that calcium induced only the changes in lipid mobility, but not hydration. Our experience shows that the relaxation time is the TDFS parameter that is the most sensitive to the adsorption of salt ions, while hydration changes are detected only rarely. When paired with computer simulations changes in TDFS relaxation time usually correlate with the changes in area per lipid. This is because mobility of lipids is usually more restricted in more condensed membranes. This relation, however, does not need to be true. One should be aware of the fact that it relates dynamic and structural properties of the lipid bilayer. The complex nature of calcium adsorption discussed above was not significantly affected by the presence of cholesterol in the membrane (Figure 3C, Melcrová et al., 2019). On the other hand, the change in membrane curvature resulted in qualitatively different binding pattern (Figure 3D). In highly curved membranes these are the lipid phosphates that attract the calcium ions more than carbonyls do, and these are the phosphates which mobility is hindered more than that of carbonyls. To measure these differences again the pair of Laurdan and Dtmac probes was used. Membrane curvature itself was also followed by TDFS in model zwitterionic bilayers labelled with Prodan and Patman, clearly indicating that the increased curvature elevates lipid mobility in the headgroup region (Sýkora et al., 2005). This conclusion is in full agreement with the timely results obtained by the modern label-free scattering techniques (vibrational sum frequency scattering (SFS), and second harmonic scattering (SHS)). Specifically, the combination of SFS and SHS proved the equal number of the lipid molecules in both bilayer leaflets even for the curved membranes which results in the elevated area per lipid and headgroup mobility in the outer leaflet of the bilayer (Okur et al., 2019). This effect gets more pronounced with increasing curvature. All these findings were anticipated by the TDFS approach, which further proves its potency to map the organization in the headgroup region.

## Conclusion

Within the last decade the experimental advances in label free techniques together with computational methods allowed the development of a quantitative understanding of the hydration shell of biomembranes and proteins. While most of the methods described in chapter 2 are experimentally very demanding and expensive, the application of the TDFS technique on biomolecules requires a rather simple and inexpensive TCSPC equipment. The TDFS technique is a robust experimental technique with a remarkable reproducibility. The read-out parameters 1) overall dynamic Stokes shift Δν and 2) characteristic relaxation time τ can report directly (i.e. without data modelling) on subtle changes in 1) the degree of hydration and 2) mobility, respectively, of the hydrated phospholipid or protein segment at the close vicinity of the fluorophore embedded in the bilayer. This implies that for a meaningful application of the TDFS technique the precise knowledge on the location of the dye is required. In protein science this pre-requisite is achieved by site-selected labelling, while the location of the chromophore of amphiphilic membrane probes can be determined by quenching experiments and molecular dynamic (MD) simulations. Together with MD simulations the TDFS approach identified how molecular parameters like membrane curvature (Sýkora et al., 2005; Magarkar et al., 2017), lipid composition (Jurkiewicz et al., 2005; Jurkiewicz et al., 2006; Olżyńska et al., 2007; Jurkiewicz et al., 2012b; Melcrová et al., 2019), presence of ions (Vácha et al., 2010; Jurkiewicz et al., 2012b; Pokorna et al., 2013; Melcrová et al., 2016; Melcrová et al., 2019), presence of pharmaceuticals (Först et al., 2014), membrane binding of peptides (Macháň et al., 2014; Olšinová et al., 2018), or lipid oxidation (Beranova et al., 2010; Volinsky et al., 2011; Jurkiewicz et al., 2012c; Vazdar et al., 2012; Štefl et al., 2014; Kulig et al., 2015b; Kulig et al., 2016) control the hydration and mobility in the headgroup region of bilayers. On the protein side the TDFS again combined with simulations demonstrated the significance of hydration and mobility in enzyme enantioselectivity (Jesenská et al., 2009; Stepankova et al., 2013; Sykora et al., 2014) as well as demonstrated how lateral membrane pressure changes the hydration profile in transmembrane channels (Fischermeier et al., 2017).

## Glossary

2-AS: 2-(9-anthroyloxy)stearic acid
9-AS: 9-(9-anthroyloxy)stearic acid
16-AP: (16-(9-anthroyloxy)palmitoic acid
ABA-C15: N-palmitoyl-3-aminobenzanthrone
ANS: 8-Anilinonaphthalene-1-sulfonic acid
ATR-FTIR: Attenuated Total Reflectance- Fourier Transform Infrared Spectroscopy
Badan: 6-Bromoacetyl-2-Dimethylaminonaphthalene
C17DiFU: 6,8-difluoro-4-heptadecyl-7-hydroxycoumarin
CaCl2: Calcium Chloride
Chol: Cholesterol; - Coumarins: benzo-α-pyrones
Dauda: 11-((5-dimethylaminonaphthalene-1-sulfonyl)amino)undecanoic acid
Di-4-ANEPPDHQ: 1-[2-Hydroxy-3-(N,N-di-methyl-N-hydroxyethyl)ammoniopropyl]-4-[β-[2-(di-n-butylamino)-6-napthyl] vinyl]pyridinium dibromide
DMPC: 1,2-dimyristoyl-sn-glycero-3-phoshatidylcholine
DOPC: 1,2-dioleoyl-sn-glycero-3-phosphocholine
DOPE: 1,2-dioleoyl-sn-glycero-3-phosphoethanolamine
DTMAC: 4-[(n-dodecylthio)methyl]-7-(N,N-dimethylamino)-coumarin
FCS: Fluorescence Correlation Spectroscopy
FWHM: Full-Width at Half-Maxima (FWHM)
GP: Generalized Polarization
GP-FLIM: Generalized Polarization- Fluorescence Lifetime Imaging Microscopy
HLDs: Haloalkane Dehalogenases; - Laurdan: 2-dimethylamino-6-lauroyl- naphthalene
LAXS: Low Angle X-ray Scattering
MD: Molecular Dynamics
NBD: nitrobenzoxadiazole, 7-nitrobenz-2-oxa-l,3-diazol-4-y1
NMR: Nuclear Magnetic Resonance
OxPL: Oxidized Phospholipid
Patman: 6-palmitoyl-2- [[2-(triethylammonium)ethyl]methylamino]naphthalene chloride
PC: Phosphotidylcholine
PE: Phosphotidylethanolamine
PG: Phosphatidylglycerol
POPC: 1-palmitoyl-2-oleoyl-sn-glycero-3-phosphocholine
POPS: 1-palmitoyl-2-oleoyl-sn-glycero-3-phospho-L-​serine
Prodan: 6-propionyl-2-(dimethylamino) naphthalene
PS: Phosphatidylserine
QENS: Quasi-Elastic Neutron Scattering
REES: Red Edge Excitation Shift
SANS: Small Angle Neutron Scattering
SAXS: Small Angle X-ray Scattering
SFS: Sum Frequency Scattering
SHS: Second Harmonic Scattering
SR: Solvent Relaxation
TCSPC: Time-Correlated Single Photon Counting
TDFS: Time-Dependent Fluorescence Shift
TDSS: Time-Dependent Stokes Shift
THz TDS: Terahertz Time-Domain Spectroscopy
TRES: Time Resolved Emission Spectra
UV/VIS: Ultraviolet–Visible Spectroscopy
